# Fc‐gamma IIIa‐V158F receptor polymorphism contributes to the severity of Guillain‐Barré syndrome

**DOI:** 10.1002/acn3.51072

**Published:** 2020-06-02

**Authors:** Shoma Hayat, Golap Babu, Avizit Das, Zakir Hossain Howlader, Ishtiaq Mahmud, Zhahirul Islam

**Affiliations:** ^1^ Laboratory of Gut‐Brain Signaling Laboratory Sciences and Services Division (LSSD) icddr,b Dhaka 1212 Bangladesh; ^2^ Department of Biochemistry and Molecular Biology University of Dhaka Dhaka 1000 Bangladesh

## Abstract

**Objective:**

Guillain‐Barré syndrome (GBS) is a rare, life‐threatening disorder of the peripheral nervous system. Immunoglobulin G Fc‐gamma receptors (FcγRs) mediate and regulate diverse effector functions and are involved in the pathogenesis of GBS. We investigated whether the FcγR polymorphisms FcγRIIa H/R131 (rs1801274), FcγRIIIa V/F158 (rs396991), and FcγRIIIb NA1/NA2, and their haplotype patterns affect the affinity of IgG‐FcγR interactivity and influence GBS susceptibility and severity.

**Methods:**

We determined FcγR polymorphisms in 303 patients with GBS and 302 ethnically matched healthy individuals from Bangladesh by allele‐specific polymerase chain reaction. Pairwise linkage disequilibrium and haplotype patterns were analyzed based on D ´statistics and the genotype package of R statistics, respectively. Logistic regression analysis and Fisher’s exact test with corrected *P* (*P*c) values were employed for statistical comparisons.

**Results:**

FcγRIIIa‐V158F was associated with the severe form of GBS compared to the mild form (*P* = 0.005, OR = 2.24, 95% CI = 1.28–3.91; *P*c = 0.015); however, FcγR genotypes and haplotype patterns did not show any association with GBS susceptibility compared to healthy controls. FcγRIIIa‐V/V158 and FcγRIIIb‐NA2/2 were associated with recent *Campylobacter jejuni* infection (*P ≤ *0.001, OR = 0.36, 95% CI = 0.23–0.56; *P*c* ≤ *0.003 and *P* = 0.004, OR = 1.70, 95% CI = 1.18–2.44; *P*c* ≤ *0.012, respectively). Haplotype 1 (FcγRIIa‐H131R‐ FcγRIIIa‐V158F‐ FcγRIIIb‐NA1/2) and the FcγRIIIb‐NA2/2 genotype were more prevalent among anti‐GM1 antibody‐positive patients (*P* = 0.031, OR = 9.61, 95% CI = 1.24–74.77, *P*c = 0.279; *P* = 0.027, OR = 1.62, 95% CI = 1.06–2.5, *P*c = 0.081, respectively).

**Interpretation:**

FcγR polymorphisms and haplotypes are not associated with susceptibility to GBS, though the FcγRIIIa‐V158F genotype is associated with the severity of GBS.

## Introduction

Guillain‐Barré syndrome (GBS) is a post‐infectious autoimmune disorder of the peripheral nervous system that can lead to significant morbidity, long‐term disability or death.

Cross‐reactive immune responses induced by molecular mimicry between the outer core structure of infectious agents that trigger GBS and host nerve gangliosides^1^ result in a blockade of nerve conduction.^1^, ^2^ *Campylobacter jejuni* has been identified as the predominant causative microbial infectious agent in GBS.^3^, ^4^, ^5^ In addition to multifarious microorganism‐derived factors, host immunogenic factors are likely to affect GBS susceptibility as only a subset of *C. jejuni*‐infected individuals (1 in 1000–5000 cases) develop GBS.^6^, ^7^, ^8^, ^9^ Natural variations in genetic host susceptibility factors have become a focus of research on the susceptibility and severity of disease pathogenesis in GBS.

Immunoglobulin G Fc‐gamma receptors (FcγRs) are important immune‐response modulating molecules that link the cellular and humoral immune system by interacting with IgG subtypes (IgG1‐4). The most common autoantibodies in GBS are produced against GM1, GD1a and GQ1b gangliosides.^5^, ^10^, ^11^ These autoantigens may influence nerve disruption, demyelination or axonal degeneration via diverse mechanisms,^12^ including induction of inflammatory immune responses, by interacting with Fc receptors. FcγR polymorphisms can determine the vigor of inflammatory responses, affect downstream functions such as phagocytosis, antibody‐dependent cellular cytotoxicity (ADCC) and the release of inflammatory mediators, and have been implicated in the development of autoimmune disease.^13^, ^14^ Thus, FcγRs may represent important effector molecules in the pathogenesis of GBS.^15^ Three subclasses of FcγRs, namely FcγRIIa, FcγRIIIa and FcγRIIIb, exhibit allelic variation.^13^, ^16^ The most widely distributed receptor, FcγRIIa, is expressed on all types of white blood cells and has two allelic forms: FcγRIIa‐H131 and FcγRIIa‐R131. These alleles differ by the replacement of histidine by arginine at position 131 due to an A → G single nucleotide exchange at position 494.^17^, ^18^ FcγRIIa‐H131 is reported to bind human IgG2 with a higher affinity than FcγRIIa‐R131.^19^ FcγRIIIa is expressed on macrophages, dendritic cells, γ/δ T‐cells and natural killer (NK) cells.^20^ A functional polymorphism at nucleotide 559 results in either a valine (V) or phenylalanine (F) at amino acid position 158, which affects the receptor binding capacity of IgG1, IgG3, and IgG4.^21^ FcγRIIIb is expressed on neutrophils and exhibits two allelic forms, neutrophil antigen 1 (NA1) and neutrophil antigen 2 (NA2). NA1 and NA2 differ by five base substitutions (nucleotides 141, 147, 227, 277, and 349) that lead to four amino acid changes (at positions 36, 65, 82, and 106) within exon 3.^18^, ^22^ However, these allelic forms of FcγR (NA1/NA2) have different affinities for IgG1 and IgG3. Thus, the various allelic forms of FcγR may possibly determine the extent of inflammatory responses and thereby influence autoimmune diseases, including GBS.

Several studies have already evaluated the relationship between FcγR polymorphisms and the pathogenesis of GBS.^23^, ^24^, ^25^, ^26^, ^27^ FcγRIIa‐H/H131 was significantly associated with susceptibility to GBS and was also a potent risk factor for the development of GBS in a Dutch population.^23^ These findings were consistent with a study of Indian patients with GBS, but not with a report on Norwegian Caucasian patients.^24^, ^26^ One meta‐analysis indicated that every FcγRIIIb‐NA2 allele cumulatively increases the GBS severity score, though none of the genotypes or alleles were associated with susceptibility to GBS.^25^ However, consensus regarding the role of FcγR polymorphisms in the pathogenesis of GBS has not yet been established due to the inadequate statistical power of studies with small sample sizes and differences in the ethnicities of the populations tested. Thus, we aimed to evaluate whether candidate gene polymorphisms in FcγR are a major causative factor for GBS susceptibility or severity in Bangladeshi patients with *C. jejuni*‐triggered GBS, which represents the world’s largest cohort.

## Materials and Methods

### Research participants

The GBS cohort used in this study includes 303 patients with GBS (208 males, 95 females; median age: 30 years [interquartile range, 17–42]; Table 1) and 302 ethnically matched healthy controls (204 males, 99 females; median age: 34 years [interquartile range, 28–46]). Patients with GBS were diagnosed based on the previously established diagnostic criteria described by Asbury and Cornblath^28^ and enrolled from Dhaka Medical College and Hospital (DMCH), Dhaka, Bangladesh. No preference was given to race, religion, or socioeconomic status during study subject selection. Genetically unrelated healthy individuals who did not have neurological diseases, antecedent infections, recent surgery or other illnesses were included in this study following informed consent and matched with patients. Clinical, electrophysiological, and serological data were obtained from patients with informed consent.

**Table 1:** Demographic and clinical characteristics of the patients with GBS.

Characteristic	Number of patients, *n* = 303 (%)
Sex	
Male/female	208/95
Age	
Median (IQR)	30 (17–42)
Preceding illness, *n* = 303	
Diarrhea	129/303 (43)
Respiratory tract infections	45/303 (15)
Fever	25/303 (8)
Other	28/303 (9)
None/unknown	76/303 (25)
Electrophysiological classification, *n* = 247	
Axonal	146/247 (59)
Demyelinating	68/247 (27)
Unclassified	33/247 (13)
MRC sum score (at entry)	
Severely affected patients	232/303 (77)
Mildly affected patients	71/303 (23)
Serological characteristics	
Anti‐GM1‐Ab‐seropositive	118/303 (39)
*C. jejuni*‐seropositive	186/303 (61)
Disease prognosis at 6 months, *n* = 303	
Good outcome	209/303 (69)
Poor outcome	94/303 (31)

GBS, Guillain‐Barré syndrome; IQR, interquartile range; MRC, Medical Research Council; Ab, antibody; *C*. *jejuni, Campylobacter jejuni*.

Blood specimens were collected by venipuncture before patients received medication and disease outcome was evaluated by assessing clinical data at specific standard time‐points (at entry, 2 weeks, 4 weeks and 6 months). In this cohort, 75% (227/303) patients had an antecedent illness; most frequently diarrhea (43%; 129/303), followed by respiratory infection (15%, 45/303), fever (8%, 25/303) or other illnesses (9%, 28/303); 25% (76/303) of patients had history of unknown infections or no infection. Serological tests, that is, antibodies against *C. jejuni* or GM1, GD1a and GQ1b gangliosides were measured using enzyme‐linked immunosorbent assays (ELISAs).^5^, ^29^

Electrophysiological studies of 82% (247/303) of the GBS patients indicated 59% (146/247) of patients had an axonal subtype of GBS, including acute motor axonal neuropathy (AMAN) and acute motor and sensory axonal neuropathy (AMSAN), 27% (68/247) of patients had acute inflammatory demyelinating polyradiculoneuropathy (AIDP) and 13% (33/247) of cases were unclassified with inexcitable nerves or equivocal findings.^30^ Severity of disease (degree of muscle weakness) was assessed using the Medical Research Council (MRC) sum score^31^, ^32^ ranging from 0 to 60 at nadir (maximum muscle weakness); GBS patients at nadir with MRC sumscore < 40 were defined as severely affected patients and with MRC sumscore ≥ 40 were defined as mildly affected patients.^33^ The outcome of the disease was measured using the GBS disability score after 6 months of follow‐up.^34^ This study was reviewed and approved by the Institutional Review Board (IRB) and ethical committees of the icddr, b, Dhaka, Bangladesh.

### Genomic DNA isolation

Whole blood samples were collected from 605 study subjects into lithium heparin‐coated anti‐coagulation tubes for genomic DNA isolation. Genomic DNA was extracted using the QIAamp^®^ DNA Blood Midi Kit (100) (Qiagen, Hilden, Germany), dissolved in 1 × TE buffer (10 mmol/L Tris‐Cl, pH 8.0, 1 mmol/L EDTA), stored at −80°C, diluted to 10 ng/µL with Milli‐Q water and then stored at −20°C until SNP detection.

### **Fc**γ**R polymorphism detection and genotype analysis**

The FcγR polymorphisms FcγRIIa H/R131 (rs1801274), FcγRIIIa V/F158 (rs396991) and FcγRIIIb NA1/NA2 were genotyped via a previously described allele‐specific polymerase chain reaction (AS‐PCR) method using published primer sequences and reaction conditions.^18^, ^21^ Human growth hormone (*HGH*) primers (5`‐GCCTTCCCAACCATTCCCTTA‐3′ and 5′‐CTCACGGATTTCTGTTGTGTTTC‐3′) were used as an internal positive control.^18^ The PCR products were visualized on 2% agarose gels using a Molecular Imager® Gel Doc™ XR + system (Bio‐Rad Laboratories Inc).

### Statistical analysis

Statistical analysis was performed using logistic regression analysis and Fisher’s exact test with Yates' continuity correction to assess associations between the FcγR polymorphisms and disease susceptibility or subgroups. In the control group, all SNPs were within Hardy‐Weinberg equilibrium. *P* values less than 0.05 were considered statistically significant. The Bonferroni method was applied to correct the *P* values for multiple comparisons: each *P*value was multiplied by the number of comparisons and denoted *P*c (*P*c, *P* corrected). Genotype/allelic frequencies were estimated by a simple counting method and the data were processed using Microsoft Excel 2010 (Microsoft, Redmond, WA, USA), GraphPad prism (version 5.01, GraphPad software, Inc., La Jolla, CA) or SPSS (version 16.0, Company, Chicago, IL). Haplotype patterns and frequencies were analyzed using the genotype package of R statistics and their associations with GBS susceptibility and subgroups were assessed using logistic regression analysis.

## Results

### FcγRIIa, FcγRIIIa, and FcγRIIIb polymorphisms and haplotype in patients with GBS and healthy individuals

**Figure 1:**
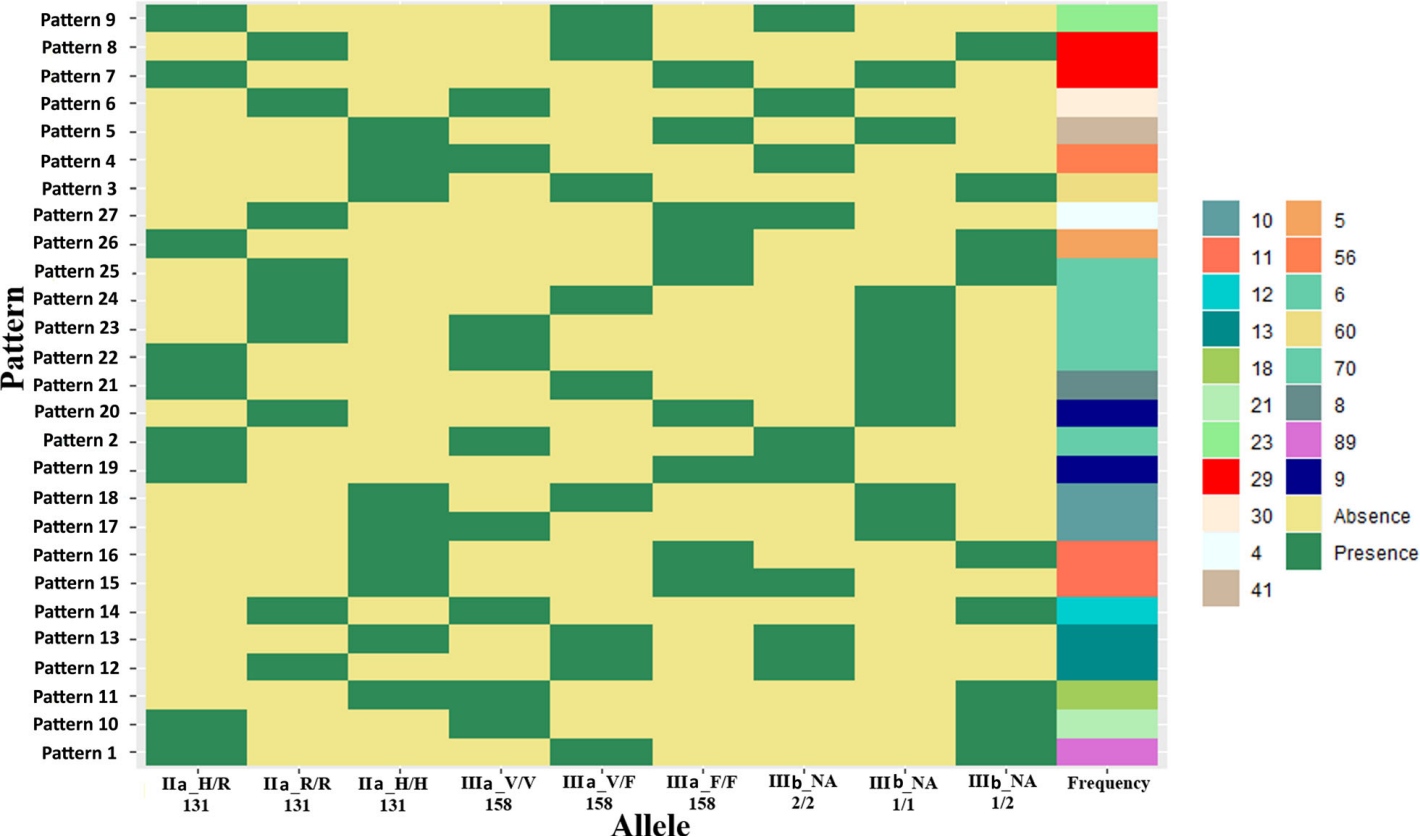
Haplotype analysis of the FcγRIIa, FcγRIIIa, and FcγRIIIb polymorphic loci for the study subjects from Bangladesh. Twenty‐seven different haplotype patterns were observed; pattern 1 was the most common (pink). Green indicates the presence and yellow indicates the absence of specific FcγR polymorphisms for each of the three loci. The polymorphism frequencies are presented as a color gradient on the right.

**Figure 2:**
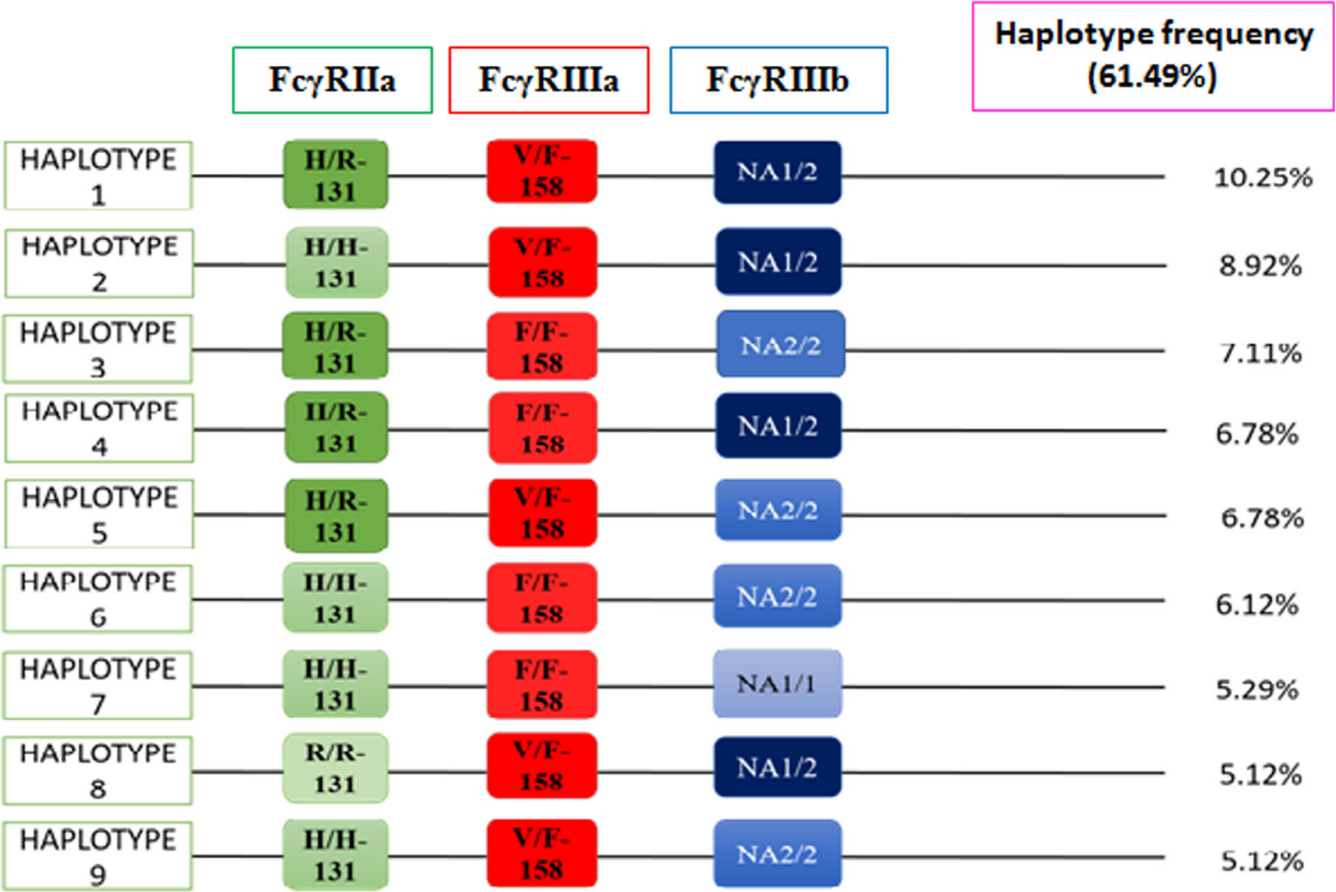
Haplotype frequencies for FcγRIIa, FcγRIIIa, and FcγRIIIb (FcγRs) polymorphisms for the study subjects from Bangladesh. The nine most predominant patterns (haplotypes 1–9; frequency >5%) represented 61.49% of total variation and were selected for haplotype analysis. The frequencies of specific haplotypes are presented on the left.

No significant associations were observed between the FcγRIIa, FcγRIIIa, and FcγRIIIb polymorphisms and susceptibility to GBS compared to healthy controls (Table 2). The comparison of axonal variants of GBS versus healthy controls or demyelinating subtypes versus healthy subjects showed no relation with disease susceptibility (Table 3). The haplotype distributions of the three loci were compared between patients with GBS and healthy individuals. Haplotype analysis revealed 27 possible different patterns for the FcγRIIa, FcγRIIIa, and FcγRIIIb polymorphic loci (Fig. 1). The nine most predominant patterns (haplotypes 1–9; frequency > 5%), representing 61.5% of total variation, were selected for further haplotype analysis (Fig. 2). No significant association was observed between any haplotype and GBS susceptibility when each haplotype was analyzed individually.

**Table 2:** FcγR genotype and allelic distributions in Bangladeshi patients with GBS and healthy controls.

FcγR genotype/allele	HC, *n* = 302 (%)	GBS patients, *n* = 303 (%)	*P* value	Odds ratio (95% CI)
FcγR‐IIa				
H/H‐131	116 (38.4)	114 (37.6)		Reference
H/R‐131	136 (45)	124 (40.9)	0.283	0.93 (0.65–1.32)
R/R‐131	50 (16.6)	65 (21.5)		1.32 (0.84–2.08)
R‐131	236 (39.1)	254 (41.9)	0.320	0.89 (0.71–1.12)
H‐131	368 (60.9)	352 (58.1)		Reference
FcγR‐IIIa				
F/F‐158	110 (36.4)	120 (39.6)		Reference
V/F‐158	150 (49.7)	143 (47.2)	0.723	0.87 (0.62–1.23)
V/V‐158	42 (13.9)	40 (13.2)		0.87 (0.53–1.45)
V‐158	234 (38.7)	223 (36.8)		1.09 (0.86–1.37)
F‐158	370 (61.3)	383 (63.2)	0.514	Reference
FcγR‐IIIb				
NA1/1	69 (22.9)	56 (18.5)		Reference
NA1/2	126 (41.7)	125 (41.2)	0.311	1.22 (0.79–1.88)
NA2/2	107 (35.4)	122 (40.3)		1.41 (0.91–2.18)
NA1	264 (43.7)	237 (39.1)	0.115	1.21(0.96–1.52)
NA2	340 (56.3)	369 (60.9)		Reference

GBS, Guillain‐Barré syndrome; HC, healthy controls; 95% CI, 95% confidence interval.

**Table 3:** Distribution of FcγR genotypes and alleles among axonal and demyelinating cases of GBS compared to healthy controls.

FcγR	Subtype			Axonal versus HC		Demyelinating versus HC	
Genotypes/Alleles	Axonal, *n* = 146 (%)	Demyelinating, *n* = 68 (%)	Healthy control (HC), *n* = 302 (%)	*P* value	OR (95% CI)	*P* value	OR (95% CI)
FcγR IIa							
H/H −131	50 (34.2)	28 (41.2)	116 (38.4)		Reference		Reference
H/R‐131	63 (43.2)	24 (35.3)	136 (45)	0.289	1.1 (0.69–1.68)	0.242	0.7 (0.40–1.33)
R/R −131	33 (22.6)	16 (23.5)	50 (16.6)		1.5 (0.88–2.66)		1.3 (0.66–2.67)
R‐131	129 (44.2)	56 (41.2)	236 (39.1)		Reference		Reference
H‐131	163 (55.8)	80 (58.8)	368 (60.9)	0.147	1.2 (0.93–1.64)	0.698	1.1 (0.75–1.59)
FcγR IIIa							
F/F‐158	57 (39)	33 (48.5)	110 (36.4)		Reference		Reference
V/F‐158	74 (50.7)	27 (39.7)	150 (49.7)	0.542	0.9 (0.6–1.4)	0.178	0.6 (0.3–1.0)
V/V‐158	15 (10.3)	8 (11.8)	42 (13.9)		0.7 (0.4–1.3)		0.6 (0.3–1.5)
V‐158	104 (35.6)	43 (31.6)	234 (38.7)		Reference		Reference
F‐158	188 (64.4)	93 (68.4)	370 (61.3)	0.378	0.9 (0.65–1.17)	0.141	0.7 (0.49–1.09)
FcγR IIIb							
NA1/1	27 (18.5)	17 (25)	69 (22.8)		Reference		Reference
NA1/2	61 (41.8)	25 (36.8)	126 (41.7)	0.506	0.8 (0.5–1.4)	0.753	1.2 (0.6–2.4)
NA2/2	58 (39.7)	26 (38.2)	107 (35.4)		0.7 (0.4–1.2)		1.0 (0.5–2.0)
NA1	115 (39.4)	59 (43.4)	264 (43.7)		Reference		Reference
NA2	177 (60.6)	77 (56.6)	340 (56.3)	0.248	0.8 (0.6–1.1)	1.0	1.0 (0.7–1.4)

OR, odds ratio; 95% CI, 95% confidence interval.

### FcγRIIa, FcγRIIIa, and FcγRIIIb polymorphisms and haplotypes in anti‐GM1 antibody‐positive GBS

The frequency of FcγRIIIb‐NA2/2 genotypes was predominant among anti‐GM1 antibody‐positive patients compared to healthy individuals but association was not significant (*P* = 0.051, OR = 1.93, 95% CI = 1.03–3.62; Table 4). Haplotype 1 (FcγRIIa‐H131R‐ FcγRIIIa‐V158F‐ FcγRIIIb‐NA1/2) and the FcγRIIIb‐NA2/2 genotype were significantly prevalent among anti‐GM1 antibody‐positive patients than antibody‐negative patients with GBS; however, these associations were lost after Bonferroni correction (*P* = 0.031, OR = 9.61, 95% CI = 1.24–74.77; *P*c = 0.279 and *P* = 0 .027, OR = 1.62, 95% CI = 1.06–2.5; *P*c = 0.081; respectively; Table 5). The homozygous FcγRIIIb NA1/1 genotype was predominant in healthy individuals compared to anti‐GM1 antibody‐positive patients (22.9% vs. 14.2%; Table 4) and significantly present in anti‐GM1 antibody‐negative patients with GBS than antibody‐positive patients (*P* = 0.002, OR = 0.43, 95% CI = 0.25–0.73; *P*c = 0.006; Table 5). Except haplotype 1, no other haplotypes (haplotype 2–9) were associated with anti‐GM1 antibody positivity (Table 5).

**Table 4:** Distribution of FcγR genotypes and alleles between healthy controls versus *C. jejuni‐*seropositive patients and healthy controls versus Anti‐GM1 antibody‐seropositive patients with GBS.

FcγR genotype/allele	Healthy controls (a), *n* = 302 (%)	*C. jejuni* seropositivepatients (b), *n* = 186 (%)	Anti‐GM1‐Ab‐seropositive patients (c), *n* = 119 (%)	a versus b *P* value	a versus b *P* corrected (*P*c)	Odds ratio (95% CI)	a versus c *P* value	a versus c *P* corrected (*P*c)	Odds ratio (95% CI)
FcγR‐IIa									
H/H‐131	116 (38.4)	67 (36.0)	42 (35.3)			Reference			Reference
H/R‐131	136 (45)	81 (43.6)	53 (44.5)	0.917	*na*	1.03 (0.69–1.55)	0.809	*na*	1.08 (0.67–1.73)
R/R‐131	50 (16.6)	38 (20.4)	24 (20.2)	0.351	*na*	1.32 (0.78–2.21)	0.354	*na*	1.33 (0.73–2.42)
R‐131	236 (39.1)	157 (42.2)	101 (42.4)			0.88 (0.68–1.14)			0.87 (0.64–1.18)
H‐131	368 (60.9)	215 (57.8)	137 (57.6)	0.347	*na*	Reference	0.391	*na*	Reference
FcγR‐IIIa									
F/F‐158	110 (36.4)	70 (37.6)	44 (37.3)			Reference			Reference
V/F‐158	150 (49.7)	90 (48.4)	55 (46.6)	0.839	*na*	0.94 (0.63–1.40)	0.722	*na*	0.92 (0.57–1.46)
V/V‐158	42 (13.9)	26 (14.0)	20 (16.1)	1.0	*na*	0.97 (0.55–1.73)	0.623	*na*	1.20 (0.63–2.25)
V‐158	234 (38.7)	142 (38.2)	95 (39.9)			1.02 (0.78–1.34)			0.95 (0.70–1.29)
F‐158	370 (61.3)	230 (61.8)	143 (60.1)	0.892	*na*	Reference	0.754	*na*	Reference
FcγR‐IIIb									
NA1/1	69 (22.9)	27 (14.3)	17 (14.2)			Reference			Reference
NA1/2	126 (41.7)	86 (46.2)	51 (42.9)	0.041	0.123	1.74 (1.03–2.94)	0.134	*na*	1.64 (0.88–3.06)
NA2/2	107 (35.4)	73 (39.3)	51 (42.9)	0.048	0.144	1.74 (1.02–2.98)	0.051	*na*	1.93 (1.03–3.62)
NA1	264 (43.7)	140 (37.6)	85 (35.7)			1.29 (0.98–1.68)			1.40 (1.02–1.91)
NA2	340 (56.3)	232 (62.4)	153 (64.3)	0.071	*na*	Reference	0.036	0.072	Reference

OR, odds ratio; 95% CI, 95% confidence interval; *C. jejuni, Campylobacter jejuni;* Anti‐GM1 Ab, Anti‐GM1 antibody; *na,* not applicable.

**Table 5:** Associations between FcγR genotypes and haplotypes with severe disease, anti‐GM1 antibody‐seropositivity and *C. jejuni*‐seropositivity among patients with GBS.

Variables	FcγR genotype/haplotype	*P* value	Odds ratio	95% CI	*P* corrected (*P*c)
Mildly affected (*n* = 71) versus severely affected (*n* = 232) patients	FcγRIIIa				
	F/F‐158	0.03	0.55	0.32–0.94	0.09
	V/F‐158	0.005	2.24	1.28–3.91	0.015
	V/V‐158	0.25	0.68	0.32–1.41	–
	FcγRIIIb				
	NA1/1	0.007	0.41	0.22–0.77	0.021
	NA1/2	0.054	1.75	0.99–3.08	0.162
	NA2/2	0.891	1.06	0.62–1.82	–
Anti‐GM1 Ab‐seropositive (*n* = 118) versus seronegative (*n* = 185)	FcγRIIIb				
	NA1/1	0.002	0.43	0.25–0.73	0.006
	NA1/2	0.482	1.16	0.76–1.77	–
	NA2/2	0.027	1.62	1.06–2.47	0.081
	Haplotype 1	0.031	9.61	1.24–74.77	0.279
*C. jejuni*‐seropositive (*n* = 186) versus seronegative (*n* = 117)	FcγRIIIa				
	F/F‐158	0.038	1.47	1.02–2.11	0.114
	V/F‐158	0.025	1.49	1.05–2.10	0.025
	V/V‐158	≤0.001	0.36	0.23–0.56	≤0.003
	FcγRIIIb				
	NA1/1	≤0.001	0.32	0.21–0.49	≤0.003
	NA1/2	0.026	1.48	1.05–2.10	0.078
	NA2/2	0.004	1.70	1.18–2.44	0.012

OR, odds ratio; 95% CI, 95% confidence interval; MRC sum scores < 40 at nadir were defined as severely affected; MRC sum scores ≥ 40 were defined as mildly affected^33^; Anti‐GM1 Ab, Anti‐GM1 antibody; *P*c, Bonferroni‐corrected *P* values.

### Associations of FcγRIIa, FcγRIIIa, and FcγRIIIb polymorphisms and haplotype patterns with disease severity and outcome

FcγRIIa, FcγRIIIa, and FcγRIIIb genotypes and haplotype patterns were investigated in patients with severe and mild form of GBS (Table 5). The haplotype patterns were not associated with disease severity, though homozygous FcγRIIIa‐F158 was significantly associated with the mild form of disease before Bonferroni correction (*P* = 0.03, OR = 0.55, 95% CI = 0.32–0.94; *P*c = 0.09; Table 5). Heterozygous FcγRIIIa‐V158F was significantly associated with the severe form of disease (compared to the mild form) after correcting the *P* value (*P* = 0.005, OR = 2.24, 95% CI = 1.28–3.91; *P*c = 0.015; Table 5). FcγRIIIa‐NA1/NA1 was significantly predominant in the mild form of GBS than the severe form (*P* = 0.007, OR = 0.41, 95% CI = 0.22–0.77; *P*c = 0.021; Table 5). FcγRIIIa‐NA1/NA2 tended to be more common in severe GBS (*P* = 0.054, OR = 1.75, 95% CI = 0.99–3.08; *P*c = 0.162; Table 5). However, the FcγRIIa‐H131 and FcγRIIa‐R131 alleles and genotypes were not associated with the severity of GBS. Individual FcγR genotypes were not associated with disease outcome at 6‐month follow‐up.

### FcγRIIa, FcγRIIIa, and FcγRIIIb genotypes in patients with recent *C. jejuni* infection

The homozygous FcγRIIIb‐NA2 and heterozygous FcγRIIIb‐NA1/2 genotypes were associated with recent *C. jejuni* infection in patients with GBS; however, the association for the heterozygous FcγRIIIb‐NA1/2 genotype lost significance after Bonferroni correction (*P* = 0.004, OR = 1.70, 95% CI = 1.18–2.44; *P*c = 0.012 and *P* = 0.026, OR = 1.48, 95% CI = 1.05–2.10; *P*c = 0.078; respectively; Table 5). Frequency of homozygous FcγRIIIb‐NA2 and heterozygous FcγRIIIb‐NA1/2 genotypes were significantly prevalent in *C. jejuni* infected patients with GBS compared to healthy controls. But *P‐*value lost its significance after Bonferroni correction (*P* = 0.041, OR = 1.74, 95% CI = 1.03–2.94; *P*c = 0.123 and *P* = 0.048, OR = 1.74, 95% CI = 1.02–2.98; *P*c = 0.144; respectively; Table 4). The FcγRIIIa‐V/V158 genotype was less frequent in *C. jejuni* ‐seropositive patients (*P ≤ *0.001, OR = 0.36, 95% CI = 0.23–0.56; *P*c ≤ 0.003; Table 5); however, the FcγRIIIa‐F/F158 and FcγRIIIa‐V/F158 genotypes were significantly prevalent among *C. jejuni* ‐seropositive patients than seronegative patients before correcting the *P* values (*P* = 0.038, OR = 1.47, 95% CI = 1.02–2.11; *P*c = 0.114 and *P* = 0.025, OR = 1.49, 95% CI = 1.05–2.10; *P*c = 0.075, respectively; Table 5).

## Discussion

This study investigated the association of three functionally relevant polymorphisms in FcγR and the resulting haplotype patterns with the susceptibility and severity of GBS among patients compared to healthy controls in a large cohort of GBS in Bangladesh. We found no significant associations between individual FcγR alleles or genotypes and susceptibility to GBS; however, the FcγRIIIa‐V/F158 genotype influenced the severity of disease. Moreover, associations between the FcγRIIIa and FcγRIIIb genotypes and haplotype patterns were evident in patients with an antecedent *C. jejuni* infection and anti‐GM1 antibody‐positive patients, respectively.

Associations between FcγR polymorphisms and susceptibility to GBS have previously been studied in patients with different ethnic backgrounds (Table 6).^23^, ^24^, ^25^, ^26^ We observed no significant differences in the FcγR allele or genotype frequencies and haplotype patterns between Bangladeshi patients with GBS and healthy controls. These findings confirm a previous meta‐analysis of British, Dutch, and Norwegian GBS cases,^25^ which suggested FcγR polymorphisms were not related to disease susceptibility, regardless of ethnic variation.

**Table 6:** Summary of population‐association studies of Fc‐gamma receptor polymorphisms with GBS disease susceptibility and severity in various ethnicities.

Study (Author, year)	Ethnic origin/population	Country	Participants (*n*) (GBS vs. controls)	Reported association
van der Pol WL, 2000	Caucasian	Netherlands	31 versus 187	FcγRIIa‐H/H131 more frequent in patients than controls (OR, 2.45; *P* = 0.037).
				FcγRIIa‐H/H131 associated with disease severity (OR, 18.57; *P* = 0.007).
Vedeler, 2000	Caucasian	Norway	62 versus 89	FcγRIIIb‐NA1/NA1 associated with mild GBS (*P* = 0.027).
van Sorge, 2005	Caucasian	Netherlands	192 versus 514	FcγRIIIb‐NA2/2 more frequent in severe GBS (OR, 2.03; *P* = 0.03).
van Sorge, 2005	British	United Kingdom	91 versus 111	FcγRIIa‐H/H131 more frequent in patients than controls (OR, 2.48; *P* = 0.02)
				FcgRIIIa‐F158 allele more frequent in patients than controls (OR, 1.56; *P* = 0.03).
Sinha, 2010	Asian	India	80 versus 80	FcγRIIa‐H/H131 and FcγRIIa‐H131 more frequent in patients than controls (*P ≤ *0.0001 and *P ≤ *0.0001)
				FcγRIIIa‐V/V158 more frequent in patients than controls (*P ≤ *0.0001)
This study	Asian	Bangladesh	303 versus 302	FcγRIIIa‐V/F158 associated with severe GBS (OR, 2.24; *P* = 0.015). FcγRIIIb NA1/NA1 associated with mild GBS (OR, 0.41; *P* = 0.02)

GBS, Guillain‐Barré syndrome; OR, odds ratio.

In addition, we found the FcγRIIIa‐F/F158 genotype was associated with the mild form of GBS based on MRC sum score at nadir, while the FcγRIIIa‐V/F158 genotype was associated with the severe form of GBS. As phagocytosis, cellular cytotoxicity, cytokine production, and other immune responses depend on efficient FcγR‐IgG interactions, the higher frequency of FcγRIIIa‐F/F158 among patients with the mild form of GBS may indicate this genotype reduces the affinity of IgG binding and in turn impairs immune complex clearance and decreases subsequent inflammation.^13^, ^35^, ^36^ Patients with FcγRIIIa‐V/F158 genotypes may have better ability to clear immune complexes (ICs) via degranulation and phagocytosis more efficiently, resulting in more severe disease.^36^ We observed a higher frequency of FcγRIIIb‐NA1/NA1 genotypes in patients with the mild form of GBS, similar to a previous study of Norwegian patients with GBS.^24^ The NA1/NA1 genotype has a high affinity for IgG1 and IgG3,^37^ which are the most common among the anti‐GM1 and anti‐GQ1b antibodies.^38^ Autoantibodies such as anti‐ganglioside antibodies are neutralized in the circulation, thus cross‐reaction of these auto‐antibodies with the peripheral nerves may be partially prevented in patients with GBS who are homozygous for FcγRIIIb‐NA1.^24^

Ganglioside‐specific IgG have been reported to damage nerve tissues by activating effector functions (eg, phagocytosis and/or degranulation) via FcγR.^35^, ^39^ Homozygous FcγRIIIb‐NA1 was less frequent among both *C. jejuni* ‐seropositive patients and anti‐GM1 antibody‐positive patients with the mild form of the disease. In contrast, FcγRIIIb‐NA2/2 was associated with recent *C. jejuni* infection and anti‐GM1 antibody production. In addition, *C. jejuni ‐*seropositive patients had higher frequencies of the FcγRIIIa‐F/F158 and FcγRIIIa‐V158F genotypes. These findings indicate *C. jejuni ‐*seropositive patients with higher frequency of the FcγRIIIa‐V158F genotype may suffer severe muscle weakness.

One limitation of this study is that polymorphisms of FcγRIIIb receptor gene, FcγRIIIb‐SH alleles were not investigated; however, it is not yet known whether FcγRIIIb‐SH polymorphisms influence the function of FcγRIIIb or not.^16^, ^40^

The present study strengthens the evidence that FcγR polymorphisms and haplotypes influence the clinical and serological subgroup of GBS, as well as the strength of the immune responses that ultimately trigger the development of GBS and affect disease severity. In addition, the FcγRIIIa‐V158F genotype was more frequent among patients with recent *C. jejuni* infection and was found to contribute to disease severity. Variation in the FcγR gene differs greatly between populations of different ethnicities, thus it will be important and interesting to confirm our findings in a multiethnic population, such as the International GBS Outcome Study (IGOS) population.^41^

## Authors’ Contributions

ZI and SH conceived and designed the study. SH and MGB contributed to data acquisition. SH, MGB, and AD performed data analysis and interpreted the data. ZI and SH drafted the manuscript, which was critically reviewed by MGB, AD, ZHH, and IM for intellectual content. All authors read and approved the final manuscript before submission.

## Conflict of Interest

ZI received funding from the Fogarty International Center, National Institute of Neurological Disorders and Stroke of the National Institutes of Health, USA under Award Number K43 TW011447) and Annexon Biosciences (South San Francisco, CA 94080, USA). SH, MGB, AD ZHH and IM have no conflicts of interest to declare.
